# Multi-walled carbon nanotubes/carbon black/rPLA for high-performance conductive additive manufacturing filament and the simultaneous detection of acetaminophen and phenylephrine

**DOI:** 10.1007/s00604-023-06175-2

**Published:** 2024-01-15

**Authors:** Robert D. Crapnell, Iana V. S. Arantes, Jéssica R. Camargo, Elena Bernalte, Matthew J. Whittingham, Bruno C. Janegitz, Thiago R. L. C. Paixão, Craig E. Banks

**Affiliations:** 1 Faculty of Science and Engineering, Manchester Metropolitan University, Chester Street, Manchester, M1 5GD UK; 2 Departmento de Química Fundamental, Instituto de Química, Universidade de São Paulo, São Paulo, SP 05508-000 Brazil; 3 Laboratory of Sensors, Nanomedicine and Nanostructured Materials, Federal University of São Carlos, Araras, 13600-970 Brazil

**Keywords:** Additive manufacturing, 3D printing, Filament production, Multi-walled carbon nanotubes (MWCNT), Differential pulse voltammetry, Carbon black, Acetaminophen, Phenylephrine

## Abstract

The combination of multi-walled carbon nanotubes (MWCNT) and carbon black (CB) is presented to produce a high-performance electrically conductive recycled additive manufacturing filament. The filament and subsequent additively manufactured electrodes were characterised by TGA, XPS, Raman, and SEM and showed excellent low-temperature flexibility. The MWCNT/CB filament exhibited an improved electrochemical performance compared to an identical in-house produced bespoke filament using only CB. A heterogeneous electrochemical rate constant, ${k}_{obs}^0$ of 1.71 (± 0.19) × 10^−3^ cm s^−1^ was obtained, showing an almost six times improvement over the commonly used commercial conductive CB/PLA. The filament was successfully tested for the simultaneous determination of acetaminophen and phenylephrine, producing linear ranges of 5–60 and 5–200 μM, sensitivities of 0.05 μA μM^−1^ and 0.14 μA μM^−1^, and limits of detection of 0.04 μM and 0.38 μM, respectively. A print-at-home device is presented where a removable lid comprised of rPLA can be placed onto a drinking vessel and the working, counter, and reference components made from our bespoke MWCNT/CB filament. The print-at-home device was successfully used to determine both compounds within real pharmaceutical products, with recoveries between 87 and 120% over a range of three real samples. This work paves the way for fabricating new highly conductive filaments using a combination of carbon materials with different morphologies and physicochemical properties and their application to produce additively manufactured electrodes with greatly improved electrochemical performance.

**Graphical abstract:**

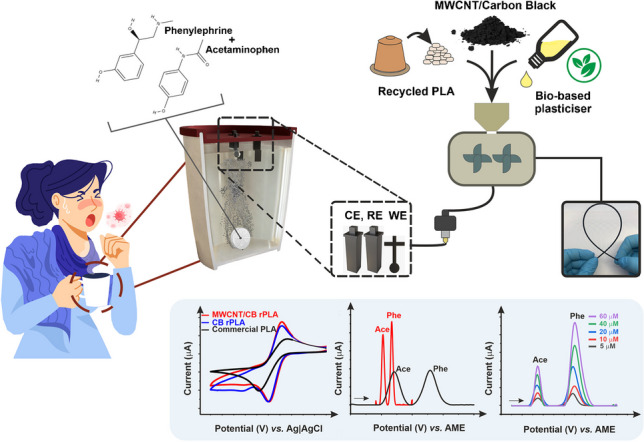

**Supplementary Information:**

The online version contains supplementary material available at 10.1007/s00604-023-06175-2.

## Introduction

Additive manufacturing (AM, 3D printing) is the generic name for a wide range of manufacturing processes that builds 3D physical objects by depositing material in a layer-by-layer process. Its popularity stems from its many advantages over the more traditional subtractive manufacturing counterparts: low (often zero) waste, on-demand production, rapid prototyping abilities, global reach, a high degree of customisability, and the ability to produce complex geometries [1]. There are many types of AM, but one with widespread adoption is fused filament fabrication (FFF), also known as fused deposition modelling (FDM). FFF functions through the extrusion of thermoplastic filament through a moving heated hot end and nozzle, which draws the cross-section of polymer specified by the print file upon the print bed or previous layer.

This form of AM has found substantial use within electrochemical research due to the overall low cost of entry and consumables. There is an increasing range of conductive filaments available commercially utilising different carbon allotropes [2], with the most widely used based on carbon black (CB) within poly(lactic acid) (PLA) [3]. A large amount of work has been reported on improving the performance of the commercial conductive PLA through either changing the printing parameters, such as printing speed [4–6] or enhancing the surface of the additively manufactured electrode through “activation” [7]. Moreover, additively manufactured and electrochemistry have been combined through the use of such commercial filaments to produce devices for water splitting [8, 9], supercapacitors [10–12], batteries [13, 14], and sensors for healthcare [15], the environment [16, 17], and forensic applications [18, 19]. This field began with printing simple discs or lollipops from commercial filament [20] but has progressed into designing full bespoke additively manufactured platforms [21], sometimes within a single print [22], and with a plethora of electrode geometries [23].

Due to the limited conductivity of the commercially available filaments, researchers have begun producing their bespoke filaments with improved electrochemical characteristics. Two main methods have been used to create bespoke filament: solvent and thermal mixing. Solvent mixing is performed by dissolving the polymer in solvents such as dichloromethane [24] or chloroform [25, 26], and the conductive filler is later dispersed in the matrix before being cast into a film, shredded, and then extruded. This method can facilitate a high loading of carbon filler but can struggle from uneven dispersion, a long production time, and polymer degradation due to chemical and thermal mixing methods. For thermal mixing, the components of the filament are added to a heated chamber and mixed with rotary blades for a specified time, creating an even dispersion of filler throughout the polymer. This method requires the inclusion of an additional plasticiser, which helps to stabilise the mix and gives the resultant filament an excellent low-temperature flexibility. This method uses no solvents, gives excellent dispersion of filler, significantly reduces the production time, and improves the environmental impact of the process [27, 28].

Most reported bespoke filaments have focussed on incorporating a single filler to induce conductivity, such as carbon black, graphite, or graphene. These carbon allotropes have different morphologies, meaning significantly different properties can be seen between these filaments. More recently, a mixed material filament has been reported combining carboxylated multi-walled carbon nanotubes (MWCNT) with CB to produce a biosensor toward yellow fever [29]. This filament was produced using thermal mixing for 10 min with poly(ethylene succinate) added as a plasticiser and reports that the increased amount of carboxyl groups on the surface of the MWCNT helps with the immobilisation of high-quantities of antibodies. However, when surface modification of the additively manufactured electrodes is not required, using specific carboxylated MWCNT is unnecessary. The combination of CB and MWCNT has also been reported in a polypropylene polymer melt, where the formation of grape-cluster-like networks offered to enhance the efficiency of the conductive pathways [30].

In this work, we propose for the first time, the incorporation of MWCNT and CB within a recycled PLA feedstock, using castor oil as a bio-based plasticiser [27], to produce a high-performance conductive additively manufactured filament with enhanced sustainability. The synergy of MWCNT and CB aims to increase the conductivity throughout the polymer matrix by forming characteristic cluster-like morphologies. The sustainability of producing this filament is enhanced through the use of recycled PLA, a bio-based plasticiser, and solvent-free melt mixing of the composite.

## Experimental section

### Chemicals

All chemicals used were of analytical grade and used as received without any further purification. All solutions were prepared with deionised water of resistivity not less than 18.2 MΩ cm from a Milli-Q Integral 3 system from Millipore UK (Watford, UK). Hexaamineruthenium (III) chloride (RuHex, 98%), castor oil, potassium ferricyanide (99%), potassium ferrocyanide (98.5–102%), sodium hydroxide (>98%), potassium chloride (99.0–100.5%), acetaminophen (≥99.0%), and phosphate-buffered saline (PBS) tablets were purchased from Merck (Gillingham, UK). (R)-Phenylephrine hydrochloride was purchased from TCI Chemicals (Zwijndrecht, Belgium). Carbon black (Super P®, >99+%) was purchased from Fisher Scientific (Loughborough, UK). Multi-walled carbon nanotubes (MWCNT, 10–30 μm length, 10–20 nm outer diameter) were purchased from Cheap Tubes (VT, USA). Recycled poly(lactic acid) (rPLA) was purchased from Gianeco (Turin, Italy). Commercial conductive PLA/carbon black filament (1.75 mm, ProtoPasta, Vancouver, Canada) was purchased from Farnell (Leeds, UK). Recycled non-conductive PLA filament was produced in-house, as shown previously [28]. Real samples of pharmaceuticals Max Strength Cold & Flu Capsules (Optipharma), Max Strength Cold & Flue Relief powder (Sainsbury’s), and Cold & Flu Powder (Beechams) were obtained from local convenience stores.

### Recycled filament production

Prior to any mixing or filament production, all rPLA was dried in an oven at 60 °C for a minimum of 2.5 h, which removed any residual water in the polymer. The polymer composition was prepared using 65 wt% rPLA, 10 wt% castor oil, 15 wt% CB, and 10 wt% MWCNT in a chamber of 63 cm^3^. The compounds were mixed at 190 °C with Banbury rotors at 70 rpm for 5 min using a Thermo Haake Poydrive dynameter fitted with a Thermo Haake Rheomix 600 (Thermo-Haake, Germany). The resulting polymer composite was allowed to cool to room temperature before being granulated to create a finer granule size using a Rapid Granulator 1528 (Rapid, Sweden). The granulated sample was collected and processed through the hopper of a EX6 extrusion line (Filabot, VA, USA). The EX6 was set up with a single screw with four set heat zones of 60, 190, 195, and 195 °C, respectively. The molten polymer was extruded from a 1.75 mm die head, pulled along an Airpath cooling line (Filabot, VA, USA), through an inline measure (Mitutoyo, Japan), and collected on a Filabot spooler (Filabot, VA, USA). The filament was then ready to use for additive manufacturing (AM).

### Additive manufacturing of the electrodes

All computer designs and .3MF files in this manuscript were produced using Fusion 360® (Autodesk®, CA, USA). These files were sliced and converted to .GCODE files and were taken to for printing by the open-source software, PrusaSlicer (Prusa Research, Prague, Czech Republic). The additively manufactured electrodes were 3D-printed using fused filament fabrication (FFF) technology on a Prusa i3 MK3S+ (Prusa Research, Prague, Czech Republic). All additively manufactured electrodes were printed using a 0.6 mm nozzle with a nozzle temperature of 215 °C, 100% rectilinear infill, 0.15 mm layer height, and print speed of 35 mm s^−1^.

### Physiochemical characterisation

Thermogravimetric analysis (TGA) was performed using a Discovery Series SDT 650 controlled by Trios Software (TA Instruments, DA, USA). Samples were mounted in alumina pans (90 μL) and tested using a ramp profile (10 °C min^−1^) from 0 to 800 °C under N_2_ (100 mL min^−1^).

X-ray photoelectron spectroscopy (XPS) data were acquired using an AXIS Supra (Kratos, UK), equipped with a monochromated Al X-ray source (1486.6 eV) operating at 225 W and a hemispherical sector analyser. It was operated in fixed transmission mode with a pass energy of 160 eV for survey scans and 20 eV for region scans with the collimator operating in slot mode for an analysis area of approximately 700 × 300 μm, the FWHM of the Ag 3d5/2 peak using a pass energy of 20 eV was 0.613 eV. Before analysis, each sample was ultrasonicated for 15 min in propan-2-ol and then dried for 2.5 h at 65 °C as shown in our unpublished data to remove excess contamination and minimise the risk of misleading data. The binding energy scale was calibrated by setting the graphitic sp^2^ C 1s peak to 284.5 eV; this calibration is acknowledged to be flawed [31] but was nonetheless used in the absence of reasonable alternatives, and because only limited information was to be inferred from absolute peak positions.

Scanning electron microscopy (SEM) micrographs were obtained using a Crossbeam 350 Focussed Ion Beam–scanning electron microscope (FIB-SEM) (Carl Zeiss Ltd., Cambridge, UK) fitted with a field emission electron gun. Imaging was completed using a secondary electron secondary ion (SESI) detector. Samples were mounted on the aluminium SEM pin stubs (12 mm diameter, Agar Scientific, Essex, UK) using adhesive carbon tabs (12 mm diameter, Agar Scientific, Essex, UK) and coated with a 3 nm layer of Au/Pd metal using a Leica EM ACE200 coating system before imaging.

Raman spectroscopy was performed on a Renishaw PLC in Via Raman Microscope controlled by WiRE 2 software at a laser wavelength of 514 nm.

### Electrochemical experiments

All electrochemical measurements were performed on an Autolab 100N potentiostat controlled by NOVA 2.1.6 (Utrecht, the Netherlands). The electrochemical characterisation of the bespoke filament and comparison to the benchmarks were performed using a lollipop design (Ø 5 mm disc with 8 mm connection length and 2 × 1 mm thickness) electrodes alongside either an external commercial Ag|AgCl (3M KCl) reference electrode with a nichrome wire counter electrode or an additive manufactured reference electrode with an additive manufactured counter electrode. All solutions of in [Ru(NH_3_)_6_]^3+^ were prepared using deionised water of resistivity not less than 18.2 MΩ cm from a Milli-Q system (Merck, Gillingham, UK) and were purged of O_2_ thoroughly using N_2_ prior to any electrochemical experiments.

Activation of the additive-manufactured electrodes was performed before all electrochemical experiments. This was achieved electrochemically in NaOH, as described in the literature [32]. Briefly, the additively manufactured electrodes were connected as the working electrode in conjunction with a nichrome wire coil counter and Ag|AgCl (3 M KCl) reference electrode and placed in a solution of NaOH (0.5 M). Chronoamperometry was used to activate the additive manufactured electrodes by applying a set voltage of + 1.4 V for 200 s, followed by applying −1.0 V for 200 s. The additive-manufactured electrodes were then thoroughly rinsed with deionised water and dried under compressed air before further use.

## Results and discussion

Mixed carbonaceous fillers with different morphologies and dispersions are proposed to improve the effective conductive network within polymer composites. Using carbon black, which forms “grape-like” aggregates within the polymer matrix in combination with carbon nanotubes that form “branches”, could create an enhanced conductive pathway through the insulating polymer. This aim is to further improve additive manufactured platforms’ electrochemical and electroanalytical performance, making them a viable manufacturing method for reliable customised electrochemical devices.

### Production and characterisation of recycled filament

**Fig. 1:**
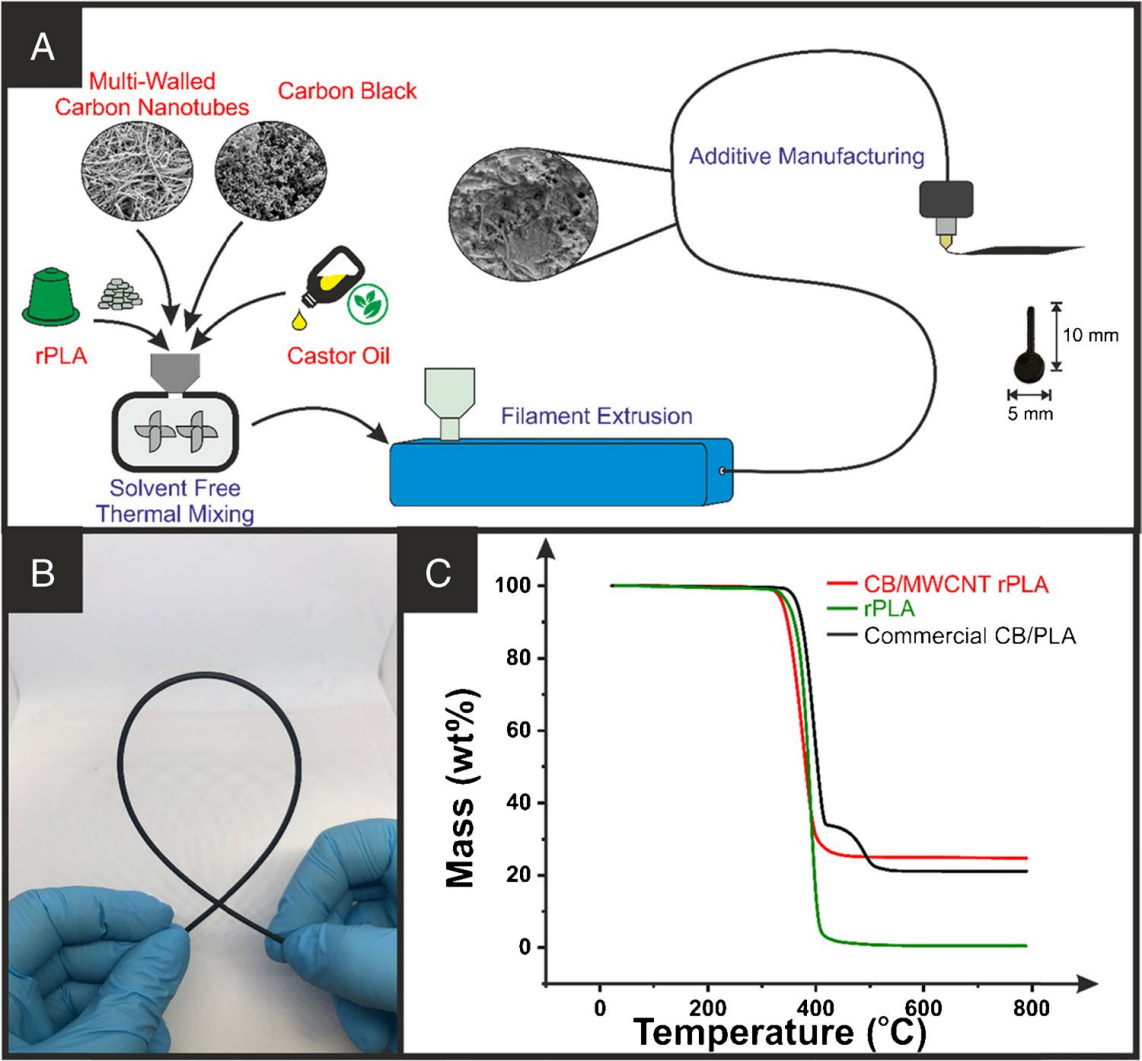
**A** Schematic for the production of bespoke conductive filament from recycled PLA, castor oil, multi-walled carbon nanotubes, and carbon black. **B** Photograph of the MWCNT/CB filament, highlighting the excellent low-temperature flexibility. **C** Thermal gravimetric analysis of the bespoke MWCNT/CB filament, commercial CB/PLA filament, and recycled PLA alone

Initial testing was performed using 25 wt% conductive filler overall as published previously [27]. Within this 25 wt%, the ratio of multi-walled carbon nanotubes (MWCNT) to carbon black (CB) was varied, summarised in Table S1, where a significant decrease in filament resistance was observed with the addition of any MWCNT. The resistance continued to decrease with increasing amounts of MWCNT; however, the printability of the filament was greatly diminished above 40% MWCNT, with the filament becoming brittle. The MWCNT with CB (10 wt%/15 wt%, 40:60% ratio of MWCNT to CB) filament was therefore chosen and then produced using the same thermal mixing techniques as outlined previously [27, 29, 33, 34] and represented in Fig. 1A. Using castor oil as the plasticiser, the structure presented in Figure S1, this methodology produced a conductive filament with excellent flexibility, as shown in Fig. 1B. This filament had a resistance measured across 10 cm of 243 ± 24 Ω, indicating that there was a substantial increase in the conductivity of the filament through mixing MWCNT and CB when compared to the results obtained previously on only CB-loaded filaments, where a resistance of (864 ± 54) Ω was measured [27] and to the commercial CB conductive filament with a quoted resistance of 2-3 kΩ over the same length [35]. Thermogravimetric analysis (TGA) of the filament, Fig. 1C, showed good agreement with previously reported data for castor oil and CB-filled rPLA [27] producing an onset temperature of degradation of (276 ± 7) °C, indicating that the composite has better thermal stability that castor oil alone. Indeed, the stabilisation of the TGA plot after the degradation of the polymeric material allowed for the confirmation of conductive filler levels within the filament to be (25 ± 1) wt%.

**Fig. 2:**
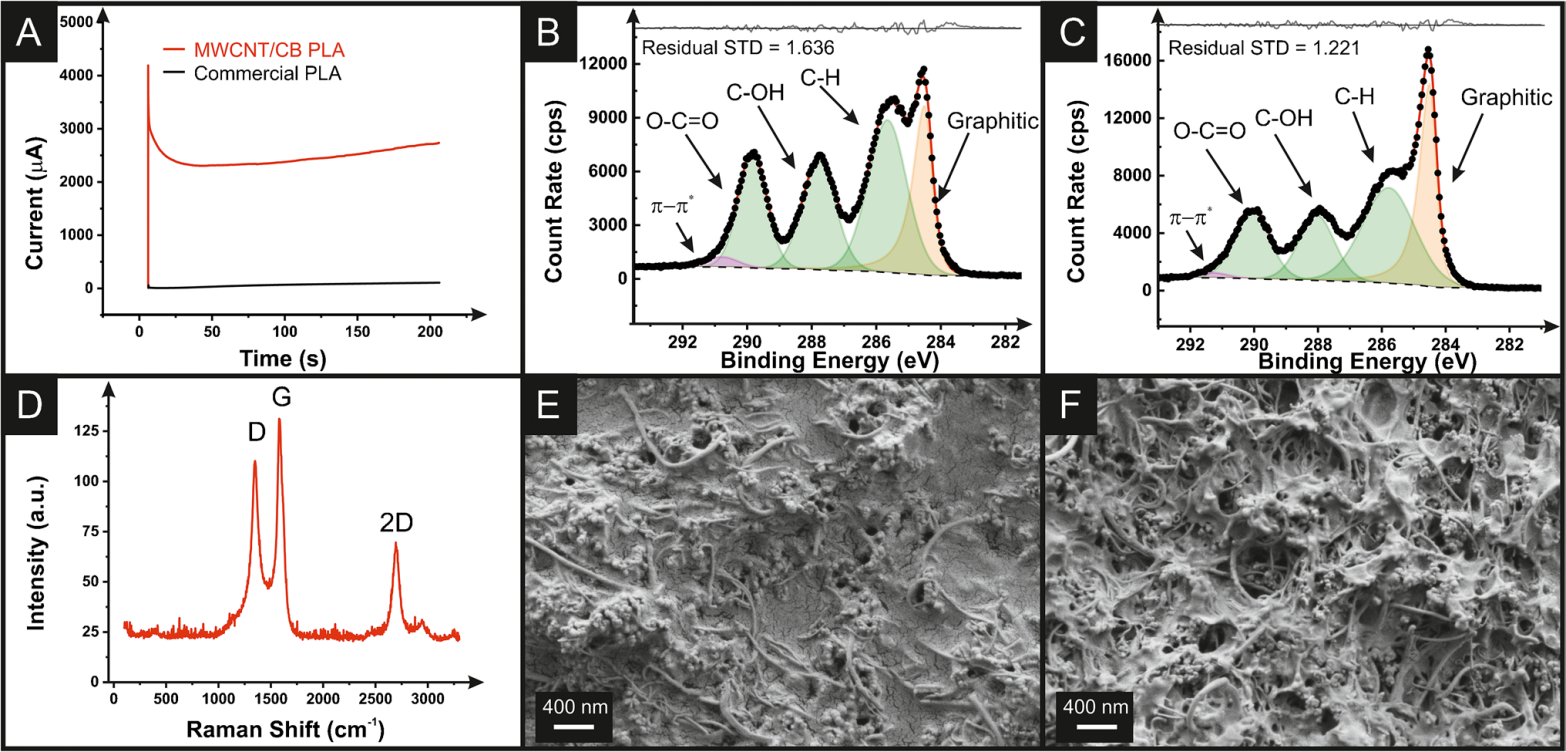
**A** Electrochemical activation profiles for the MWCNT/CB and commercial conductive PLA. The XPS C 1s region for the MWCNT/CB additively manufactured electrodes **B** before and **C** after electrochemical activation. **D** Raman spectra for activated MWCNT/CB additively manufactured electrodes. SEM images for the MWCNT/CB additively manufactured electrodes **E** before and **F** after electrochemical activation

Activation of additively manufactured electrodes is commonplace within the literature. For commercial conductive PLA filaments, it is necessary to produce a surface suitable for electrochemical experiments. However, recent work on bespoke filaments has shown that it is possible to outperform the activated additively manufactured electrodes printed from commercial filament straight from the print bed [28]. Even so, activation of these bespoke electrodes still produces an advantageous response by removing excess PLA from the surface of the electrode, exposing increased amounts of conductive material [27, 29, 36]. Aqueous electrochemical activation was performed on the MWCNT/CB additively manufactured electrodes and commercial conductive additively manufactured electrodes, Fig. 2A. In this way, the electrode was subjected to a potential of +1.4 V in sodium hydroxide (0.5 M). It can be seen that a much higher current value is obtained at the end of the 200 s compared to the commercial filament, indicating that there is an increase in the electrochemically available surface area and electrochemical performance for the MWCNT/CB electrode. To further probe this, XPS analysis was performed on the non-activated and activated additively manufactured electrode surfaces, Fig. 2B and C, respectively.

Interestingly, for adequate fitting of the non-activated additively manufactured electrodes, there was the appearance of a large asymmetric peak at 284.5 eV consistent with the X-ray photoelectron emission by graphitic carbon [37, 38]. These peaks are clearly seen on the XPS C 1s spectra obtained for the pure carbon black and MWCNT powders, Figure S2. This peak is generally not observed on non-activated samples for the commercial conductive PLA [39] or the CB-only bespoke filament [27]. An asymmetric peak at 291 eV was also required on the non-activated sample, attributed to the π-π* within the graphitic carbon [37, 38]. These features suggest that the MWCNTs are present on the surface of the additively manufactured electrodes, piercing through the plastic. For the activated sample, Fig. 2C, there is a further enhancement in the magnitude of the graphitic peak consistent with previous reports where the electrochemical activation strips surface PLA from the additively manufactured electrodes, revealing increased amounts of conductive material [27]. The atomic concentrations of the functional groups are available in Table S2 where, upon activation, there is an increase in the concentration of graphitic carbon and a reduction in the C=O and O-C=O groups. This supports the removal of polymeric material from the surface and the exposure of more graphitic carbon.

Raman analysis performed on the activated MWCNT/CB additively manufactured electrodes, Fig. 2D, showed the characteristic peaks for graphitic-like structures, with intense peaks at 1338, 1572, and 2680 cm^−1^ assigned to the D-, G-, and 2D bands, respectively [40, 41]. Moreover, the presence of the MWCNT on the surface of the non-activated and activated additively manufactured electrodes is confirmed through SEM analysis, Fig. 2E and F, respectively. On the non-activated additively manufactured electrodes, Fig. 2E, there are significant amounts of MWCNT and CB protruding from the polymer matrix, confirming the XPS results. After activation, a clear removal of polymeric material is observed (Fig. 2F), revealing an enhanced amount of MWCNTs and CB and, therefore, making this filament even more suitable for further electrochemical applications.

### Electrochemical characterisation of additively manufactured electrodes

Electrochemical characterisation of MWCNT/CB additively manufactured electrodes was performed against common outer- and inner-sphere redox probes [Ru(NH_3_)_6_]^3+^ and [Fe(CN)_6_]^4−/3−^. The results were then compared to previously reported data using the commercial conductive PLA and the bespoke CB-only rPLA [27]. A key summary of this data can be found in Table 1.

**Table 1:** Comparisons of [Ru(NH_3_)_6_]^3+^ (1 mM, 0.1 M KCl) cathodic peak currents (-I_p_^c^), peak-to-peak separations (*ΔE*_*p*_), heterogeneous electron transfer (${k}_{obs}^0$), and electrochemically active area (*A*_*real*_) for the commercial and the bespoke carbon black and MWCNT filaments. The uncertainties are the standard deviations across three different additively manufactured electrode measurements

Parameter	Commercial	CB	MWCNT/CB
-I_p_^c^ (μA)^a^	65.8 ± 3.5	86.7 ± 6.9	86.0 ± 1.0
*ΔE*_*p*_ (mV)^a^	238 ± 5	116 ± 8	111 ± 6
${k}_{obs}^0$ (cm s^−1^)^b^	0.30 (± 0.03) × 10^−3^	1.57 (± 0.18) × 10^−3^	1.71 (± 0.19) × 10^−3^
*A*_*real*_ (cm^2^)^b^	0.47 ± 0.02	0.65 ± 0.04	0.65 ± 0.05
R_S_ (Ω)^c^	650 ± 14	222 ± 10	115 ± 18
R_CT_ (kΩ)^c^	2.94 ± 0.61	0.47 ± 0.09	0.31 ± 0.07

^a^Extracted from 25 mV s^−1^ CVs; ^b^calculated using cyclic voltammetry scan rate study (5−500 mV s^−1^) in [Ru(NH_3_)_6_]^3+^ (1 mM, 0.1 M KCl); ^c^ calculated from EIS in [Fe(CN)_6_]^4−/3−^ (1 mM, 0.1 M KCl)

**Fig. 3:**
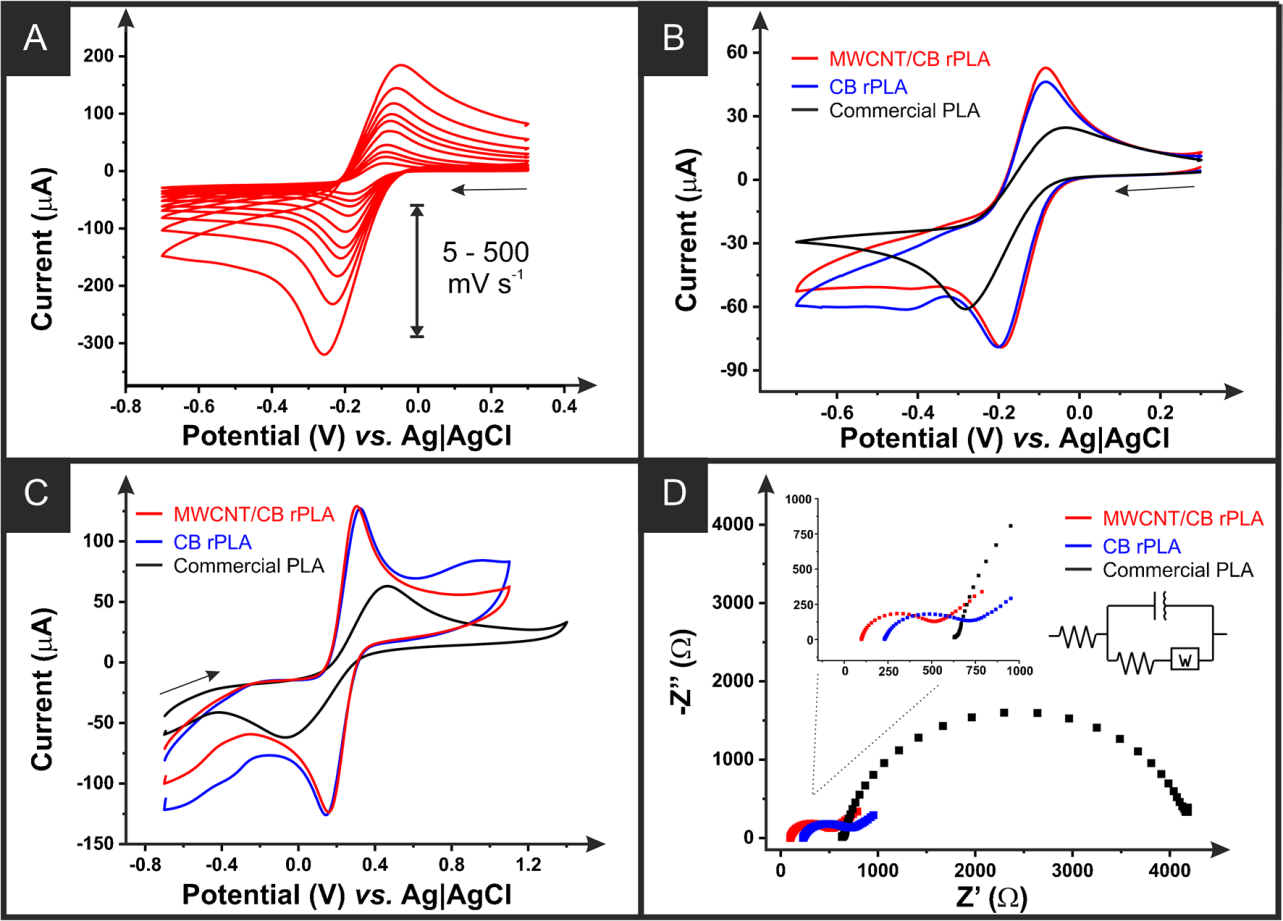
**A** Scan rate study (5−500 mV s^−1^) in [Ru(NH_3_)_6_]^3+^ (1 mM in 0.1 M KCl) performed with the MWCNT/CB filament as the WE, nichrome coil CE, and Ag|AgCl as RE. **B** Cyclic voltammogram (25 mV s^−1^) of [Ru(NH_3_)_6_]^3+^ (1 mM in 0.1 M KCl) comparing the MWCNT/CB electrode with the commercial CB/PLA and a bespoke CB/rPLA. **C** Cyclic voltammogram (25 mV s^−1^) of [Fe(CN)_6_]^4−/3−^ (1 mM in 0.1 M KCl) comparing the MWCNT/CB electrode with the commercial CB/PLA and a bespoke CB/rPLA. **D** EIS Nyquist plots of [Fe(CN)_6_]^4−/3−^ (1 mM in 0.1 M KCl) comparing the MWCNT/CB electrode with the commercial CB/PLA and CB/rPLA

Figure 3A presents an example of the scan rate study obtained for the MWCNT/CB additively manufactured electrodes within [Ru(NH_3_)_6_]^3+^ (1 mM, 0.1 M KCl), which was used for the determination of the heterogeneous electrochemical rate constant (${k}_{obs}^0$) and the real electrochemical surface area (*A*_*real*_) [42, 43]. The ${k}_{obs}^0$ of the MWCNT/CB additively manufactured electrodes was calculated to be 1.71 (± 0.22) × 10^−3^ cm s^−1^, compared to 0.30 (± 0.03) × 10^−3^ cm s^−1^ for the commercial additively manufactured electrodes and 1.57 ( ± 0.18) × 10^−3^ cm s^−1^ for the CB/PLA AME. There is also a notable increase in the electrochemical surface area for the MWCNT/CB AME over the commercial AME, confirming what is seen upon activation in Fig. 2A. Figure 3B shows the cyclic voltammogram (25 mV s^−1^) obtained against [Ru(NH_3_)_6_]^3+^ (1 mM, 0.1 M KCl) for the MWCNT/CB, CB, and commercial conductive PLA additively manufactured electrodes highlighting the improved electrochemical performance of the filament containing an additional 10 wt% MWCNT. It can be seen that there is a small additional cathodic peak at ~−0.4 V, which is proposed to be oxygen either introduced by the activation procedure or trace amounts in the solution. This is only seen in the bespoke filaments due to the enhanced kinetics of these materials in comparison to the commercial filament; however, more rigorous experimental testing is required to establish this.

To further test the electrochemical performance, cyclic voltammetry (25 mV s^−1^) was performed against the inner-sphere probe [Fe(CN)_6_]^4−/3−^ (1 mM, 0.1 M KCl), Fig. 3C. The MWCNT/CB additively manufactured electrodes again produced an enhanced performance with an increased oxidation peak current of 138 ± 9 μA, compared to 76 ± 11 μA for the commercial conductive PLA, and an improved peak-to-peak separation (*ΔE*_*p*_) of 150 ± 12 mV, compared to 494 ± 60 mV for the commercial PLA and 190 ± 12 mV for the CB/PLA. In this case, the presence of an additional anodic peak for the CB-only filament is thought to be from areas of poorly activated surface on the electrode as the presence of surface PLA hinders the electrochemical reaction for inner-sphere probes. For the additional cathodic peaks, this is again thought to be oxygen present in the sample, which is not observed for the commercial filament due to the poor kinetics of this material, although we note more experiments are required to confirm this in a future study. The Nyquist plots obtained through electrochemical impedance spectroscopy in [Fe(CN)_6_]^4−/3−^ (1 mM, 0.1 M KCl) are presented in Fig. 3D, with the corresponding Bode plots shown in Figure S3. The solution resistance (RS) and charge-transfer resistance (R_CT_) can be calculated from the equivalent circuit fitting of the Nyquist plot, with all average parameters calculated across three repeats and shown in Table S3. It can be seen that again, the MWCNT/CB filament produced an improved performance in both regards, producing an R_S_ of 115 ± 18 Ω and R_CT_ of 308 ± 65 Ω. The results obtained here show good agreement with the kinetic data obtained through cyclic voltammetric scan rate studies, whereby both bespoke filaments show significantly improved kinetics and charge-transfer resistances over those obtained by the commercial filament. Additionally, the MWNCT/CB filament shows improvements in both metrics over the only CB material [27] and a carbon black/graphite composite [44] published previously. This characterisation has shown how AM filament from recycled feedstock can be further improved in terms of conductivity and electrochemical performance by integrating mixed conductive fillers to create an enhanced conductive pathway through the polymer matrix.

### Electroanalytical determination of acetaminophen and phenylephrine

**Fig. 4:**
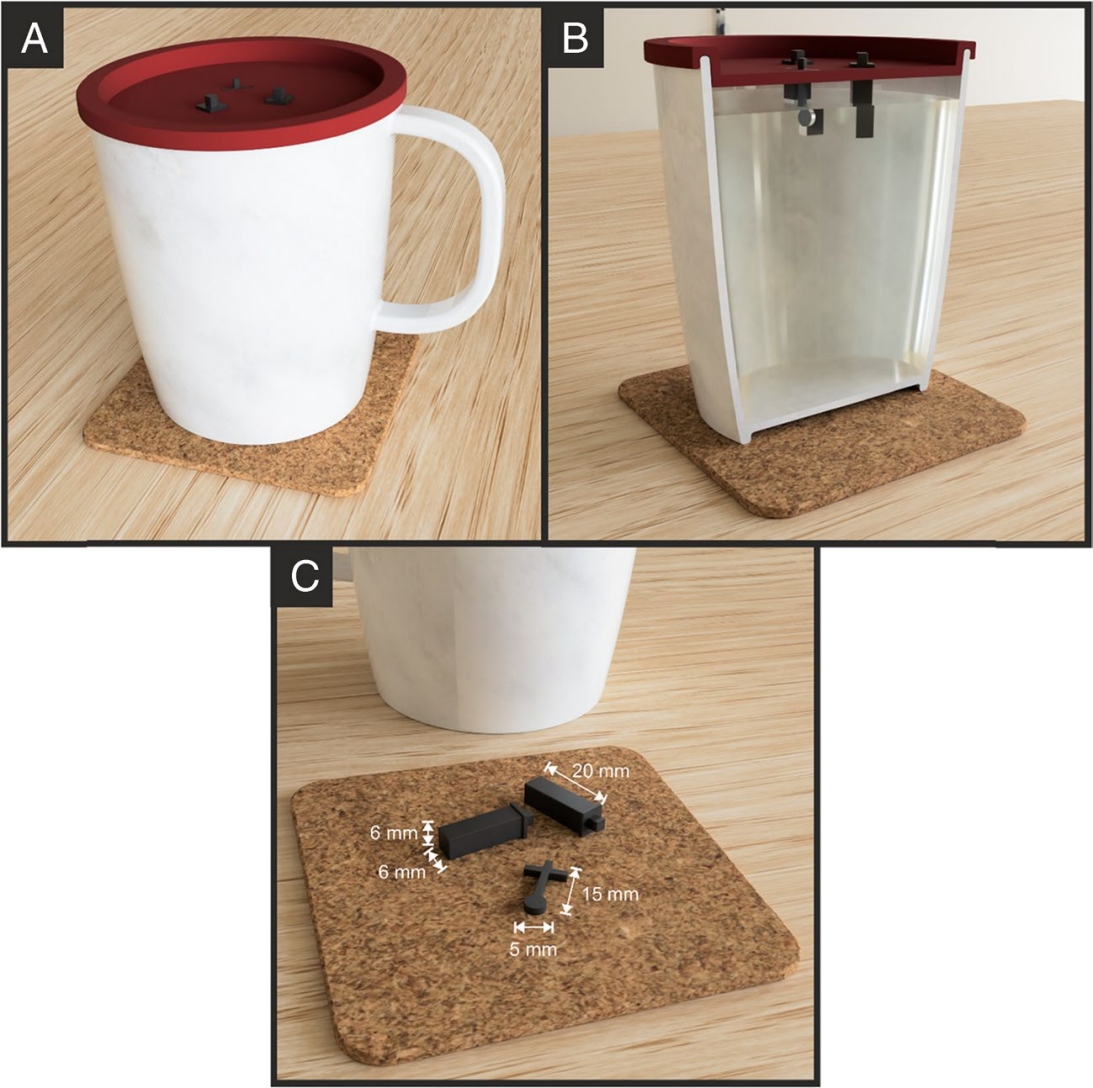
Render images of **A** the design print-at-home lid, **B** the cross section and **C** the electrodes for use on a drinking vessel

The combination of additively manufactured and electrochemistry is a powerful alternative that allows for the design of sensing platforms that can be uploaded and shared online for users to print at home and then use on demand. To demonstrate the electroanalytical capabilities of the produced MWCNT/CB filament, the simultaneous detection of acetaminophen (ACE) and phenylephrine (PHE) was performed. Table S4 shows the most important data on the calibration plots presented. The same additively manufactured electrodes were applied to detect such compounds within real pharmaceutical products commonly used at home to relieve cold and flu symptoms. To emphasise the importance of designing simple platforms that could meet non-specialised user needs, in this work, our bespoke additively manufactured electrodes were integrated onto a removable lid that could be placed onto a typical drinking vessel, a mug, or glass at home, which can be easily customised depending on the circumference of a specific vessel, Fig. 4. The lid is printed with the slots designed for the specific electrodes, which holds them in the correct position and ensures identical electrode spacing between measurements, and therefore, better reproducibility of the measurements. Please note that the reference and counter electrodes were kept identical in this design to remove any confusion over configuration. The working electrode has a cross-modified standard lollipop shape to stabilise on the designed platform.

**Fig. 5:**
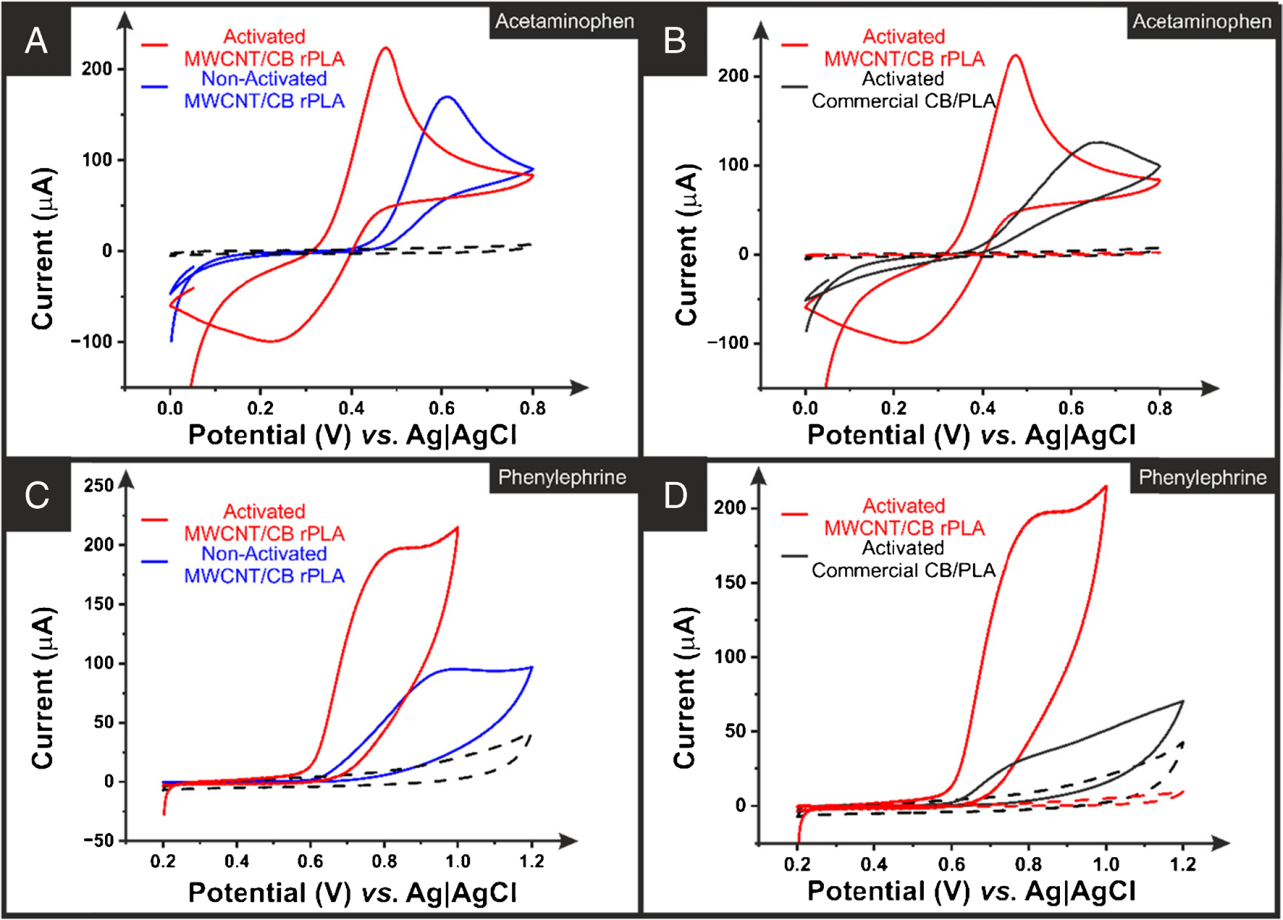
**A** Cyclic voltammograms of 0.5 mM acetaminophen in 0.01 M PBS pH 7.4 with and without activation in 0.5 M NaOH solution and **B** comparing the activated MWCNT with the commercial additively manufactured electrode using external reference and counter electrodes. **C** Cyclic voltammogram of 0.5 mM phenylephrine in 0.01 M PBS pH 7.4 with and without activation in 0.5 M NaOH solution and **D** comparing the activated MWCNT with the commercial additively manufactured electrodes using external reference and counter electrodes. Scan rate: 50 mV/s

Figure 5 shows the cyclic voltammetric (50 mV s^−1^) response for the activated and non-activated additively manufactured electrodes printed from MWCNT/CB and commercial PLA for the individual determination of phenylephrine and acetaminophen (0.5 mM in PBS, pH = 7.4). In all cases, the activated electrodes provide an expected enhanced electrochemical performance over the non-activated additively manufactured electrodes printed from the same filament. Moreover, it is key to observe in Fig. 5 that the non-activated additively manufactured electrode printed from the MWCNT/CB filament showed a significantly better electroanalytical performance when compared to the activated additively manufactured electrodes printed from commercial CB/PLA filament. For phenylephrine, the non-activated MWCNT/CB additively manufactured electrodes produced a peak current of 95.6 μA, compared to 31.6 μA for the activated commercial filament. This is observed once more for acetaminophen, where the non-activated MWCNT/CB additively manufactured electrodes produced a peak oxidation potential of +0.61 V and a peak current of 170 μA, compared to +0.65 V and 128 μA for the activated commercial filament. This underlines how the combination of MWCNT/CB within the filament can produce electrodes with a competitive conductivity and, therefore, suitable for use as-printed, with no need for additional activation, unlike the commercial CB/PLA. However, due to the activation still improving the additively manufactured electrodes, this step continued to be used throughout the electroanalytical work.

**Fig. 6:**
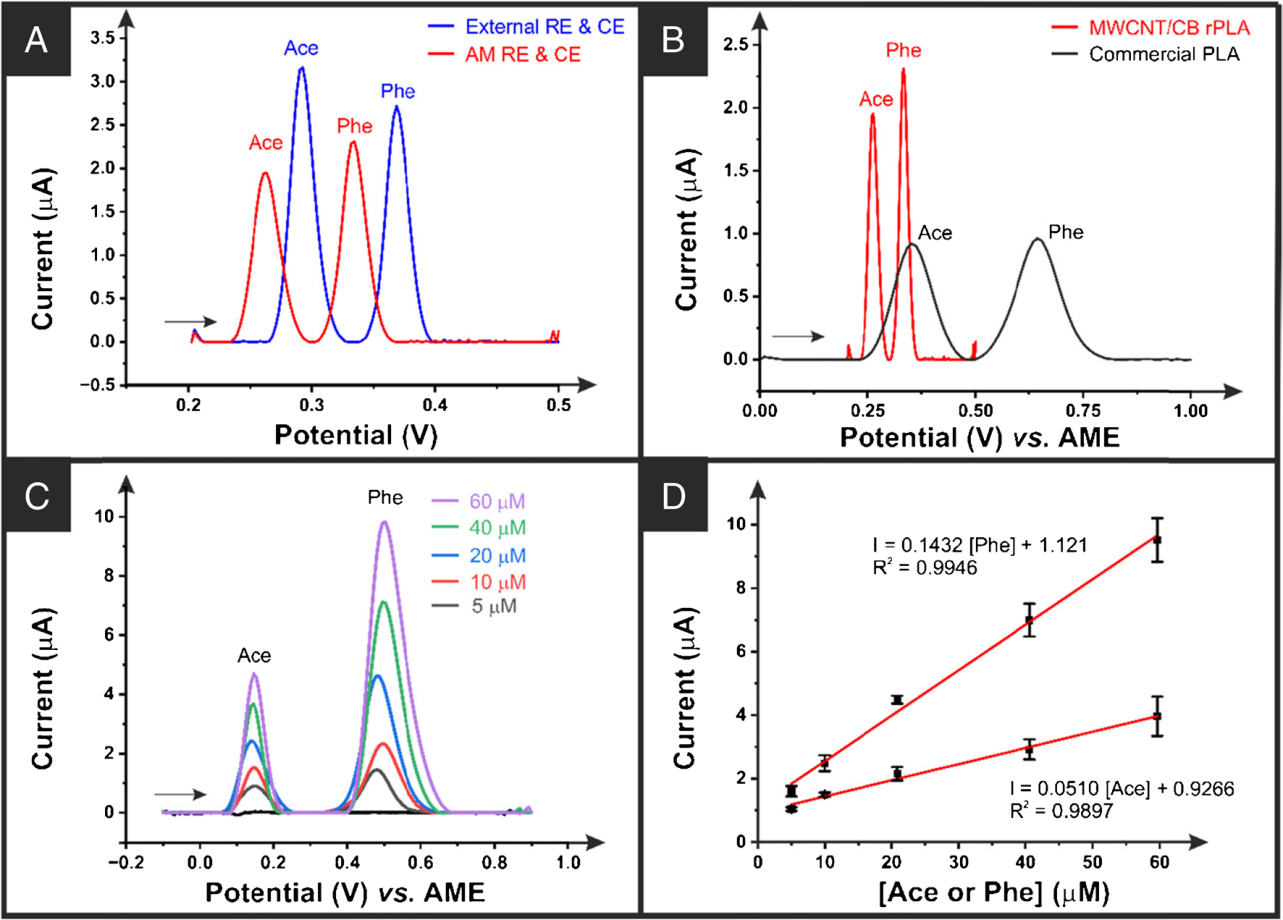
**A** Baseline corrected differential pulse voltammograms (DPVs) of a mixture of 10 μM ACE + μM PHE in 0.01 M PBS pH 7.4 using external and additively manufactured reference and counter electrodes and MWCNT additively manufactured electrode as the working electrode. **B** DPVs of a mixture of 10 μM ACE + 10 μM PHE in 0.01 M PBS pH 7.4 comparing MWCNT with the commercial additively manufactured electrode as working electrodes and using commercial additively manufactured electrode as reference and counter electrodes. **C** DPVs of acetaminophen and phenylephrine in different concentrations (5 to 60 μM) in 0.01 M PBS pH 7.4 recorded at the MWCNT/CB/rPLA using commercial additively manufactured electrode as reference and counter electrodes. Step potential 8 mV. Amplitude 50 mV. Modulation time 0.05 s. Interval time 0.5 s. **D** The respective calibration plot for simultaneous detection of ACE and PHE. Error bars are calculated from the standard deviation of three electrodes

For print-at-home sensing systems, to ensure ease of use and reduce the cost, it is advantageous to use 3D-printed reference and counter electrodes, as seen previously in the literature [22]. Figure 6A presents the differential pulse voltammetric (DPV) response for the 3-electrode platform based on MWCNT/CB additively manufactured electrodes compared to the system using only the MWCNT/CB additively manufactured electrodes as a working electrode and commercial standard reference and counter electrodes. It can be seen that there is a shift to lower peak potentials when using the additively manufactured electrodes and a reduction in peak current. However, there is still a clear separation between the two peaks for acetaminophen and phenylephrine. Figure 6B compares the response between MWCNT/CB additively manufactured electrodes and commercial conductive additively manufactured electrodes. There is a stark difference in the response, with the MWCNT/CB additively manufactured electrodes producing sharp and intense well-defined oxidation peaks at +0.26 V and +0.33 V for ACE and PHE, respectively, compared to +0.35 V and +0.64 s V for the commercial conductive additively manufactured electrodes. The peak current values demonstrate the enhanced electroanalytical performance of the MWCNT/CB filament, with a peak current of 1.95 μA and 2.31 μA for ACE and PHE, respectively, compared to 0.92 μA and 0.97 μA for the commercial conductive additively manufactured electrodes.

The print-at-home cell was then used to detect individual components in the presence of a fixed amount of the other, Figure S1. This showed that the MWCNT/CB filament could successfully detect acetaminophen in the presence of phenylephrine between 5 and 200 μM, with a sensitivity of 0.14 μA μM^−1^ and a limit of detection (LoD) of 3.3 μM. Additionally, the MWCNT/CB filament could successfully detect phenylephrine in the presence of acetaminophen between 5 and 60 μM, with a sensitivity of 0.18 μA μM^−1^ and a LoD of 7.8 μM. Throughout this work, the LoD was calculated using 3 *σ*/*m*, where *σ* is the standard deviation of the blank and *m* is the slope of the calibration plot.

Then, the simultaneous determination of acetaminophen and phenylephrine (5–60 μM) is presented in Fig. 6C, with the calibration plots shown in Fig. 6D. It can be seen that separation in the voltammetric peaks of both molecules makes reliable the simultaneous quantification. Utilising the MWCNT/CB filament, the real-time detection of acetaminophen and phenylephrine was possible with a sensitivity of 0.05 μA μM^−1^ and 0.14 μA μM^−1^, respectively. It can be seen from this that there is a decrease in the sensitivity for acetaminophen determination when simultaneous detection is performed. Even so, both chemicals were successfully determined, with LoDs for acetaminophen and phenylephrine calculated to be 0.04 μM and 0.38 μM, respectively, for the simultaneous analysis. This system was then applied to determine both compounds within three different pharmaceutical products, corresponding to one capsule and two powder drink formulations. The results obtained are summarised in Table 2, showing great recoveries in all cases. Note that experiments have been performed at low and high concentrations of acetaminophen and phenylephrine in each formulation to demonstrate the reliability of the simultaneous analysis within the linear range. We also note that interference will be possible from electroactive contaminants as no electrode modification or solution filtration/complexation has been performed, which could explain some of the larger variations in recoveries. This detection is only to provide proof of concept that print-at-home sensors for common household products will be achievable products through the development of enhanced filaments.

**Table 2:** Determination of acetaminophen (ACE) and phenylephrine (PHE) within real pharmaceutical samples. The uncertainties are the standard deviations across three different additively manufactured electrode measurements

Sample	Analyte	Added (μmol L^−1^	Detected (μmol L^−1^	Recovery (%)
Max strength capsules	PHE	40.0	50.1	125 (± 4)
	ACE	40.0	34.8	87 (± 1)
	PHE	60.0	56.2	94 (± 6)
	ACE	60.0	53.2	92 (± 2)
Max strength powder	PHE	10.0	10.1	101 (± 4)
	ACE	10.0	12.1	121 (± 1)
	PHE	40.0	48.2	121 (± 7)
	ACE	40.0	42.7	107 (± 2)
Beechams powder	PHE	5.0	5.2	103 (± 8)
	ACE	5.0	5.5	109 (± 3)
	PHE	20.0	25.6	128 (± 4)
	ACE	20.0	20.7	104 (± 11)

It is shown how additive manufacturing has the potential to produce items suitable for print-at-home sensing; however, improvements can still be made in the overall loading on the filaments, improving the sustainability of conductive carbons used and through the removal of post-processing needs such as activation. This work highlights how the production of new bespoke filaments using mixed conductive fillers can significantly improve additively manufactured electrochemical systems’ performance. This is achieved through proof-of-concept print-at-home sensors whilst still utilising recycled plastic feedstock to improve the sustainability of the systems.

## Conclusions

This work reported using MWCNT and CB in combination to produce an electrically conductive additively manufactured filament. This filament showed excellent low-temperature flexibility and significantly less resistance over a 10 cm length than a similar filament made using only CB. The additively manufactured electrodes printed from this filament were characterised through XPS, Raman, and SEM, which showed increased concentrations of graphitic carbon present on the surface of the printed parts. The additively manufactured electrodes printed from the MWCNT/CB filament showed improved electrochemical performance when compared with commercially available conductive filament and bespoke filament made from only carbon black. A print-at-home design for detecting acetaminophen and phenylephrine in pharmaceutical products was designed and successfully tested for individual and simultaneous detection. The additively manufactured electroanalytical system was then applied to determine these compounds within three real pharmaceutical products achieving good recovery values. This work highlights how using a combination of carbon materials with different morphologies can improve the conductive network created through an additively manufactured filament, resulting in an improved electrochemical performance. Additionally, we highlight how additive manufacturing and electroanalysis can synergise to produce useful devices that can be printed in situ, anywhere a low-cost 3D printer can be powered.

### Supplementary information

ESM 1 (DOCX 808 kb)
