# Supplementary material for: Multi-walled carbon nanotubes/carbon black/rPLA for high-performance conductive additive manufacturing filament and the simultaneous detection of acetaminophen and phenylephrine
Source: Mikrochim Acta. 2024 Jan 15;191(2):96. doi: 10.1007/s00604-023-06175-2 (PMC10789692; doi:10.1007/s00604-023-06175-2)
Supplement: Supplementary file 1 — (DOCX 808 kb) [file 604_2023_6175_MOESM1_ESM.docx]

**Supporting Information for:**

**Multi-walled carbon nanotubes/carbon black/rPLA for high-performance conductive additive manufacturing filament and the simultaneous detection of acetaminophen and phenylephrine**

Robert D. Crapnell,^1^ Iana V.S. Arantes,^1,2^ Jéssica R. Camargo,^1,3^ Elena Bernalte,^1^ Matthew J. Whittingham,^1^ Bruno C. Janegitz,^3^ Thiago R.L.C. Paixão,^2^ and Craig E. Banks^1*^

*^1^Faculty of Science and Engineering, Manchester Metropolitan University, Chester Street,*

*M1 5GD, United Kingdom.*

*^2^Departmento de Química Fundamental, Instituto de Química, Universidade de São Paulo, São Paulo, SP, 05508-000, Brazil.*

*^3^Laboratory of Sensors, Nanomedicine and Nanostructured Materials, Federal University of São Carlos, Araras, 13600-970, Brazil.*

^*^To whom correspondence should be addressed.

E-mail: [c.banks@mmu.ac.uk](mailto:c.banks@mmu.ac.uk); Tel: +44(0)1612471196


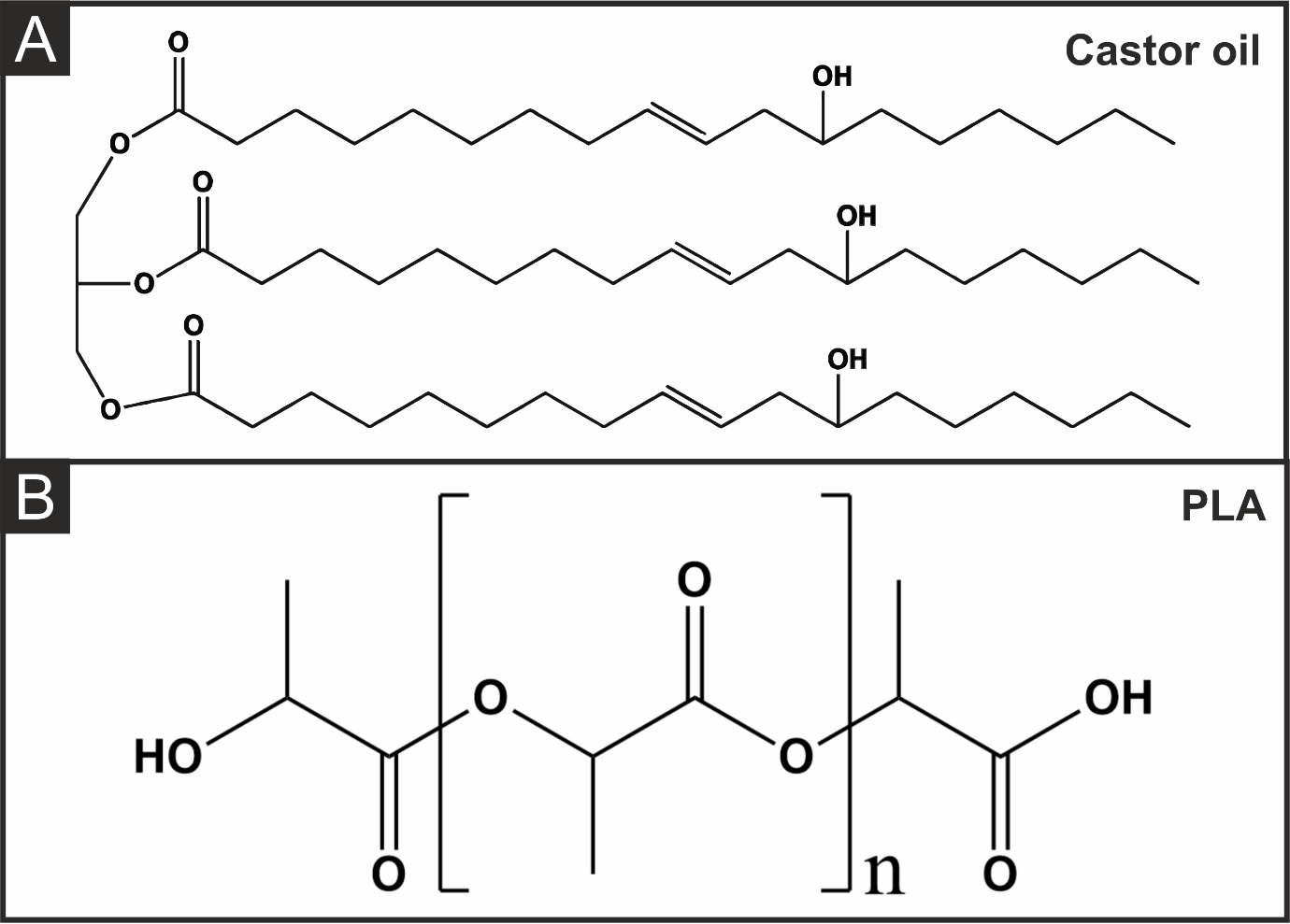


**Figure S1.** **(A)** Castor oil and **(B)** PLA structures. Reproduced with permission from ref [27]. Copyright Royal Society of Chemistry 2023.


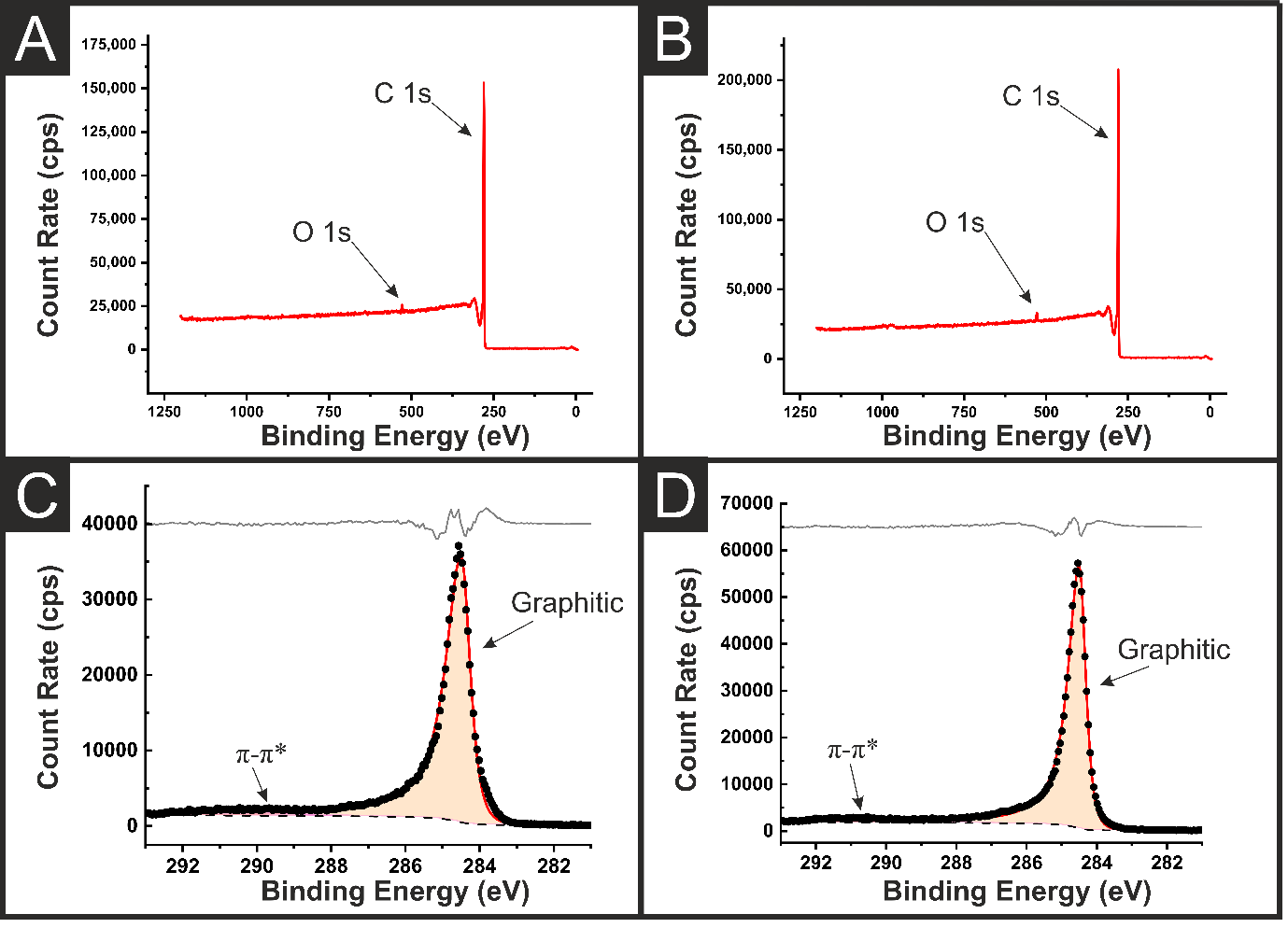
**Figure S2.** XPS wide angle spectra for (A) carbon black powder and (B) MWCNT powder, and XPS C 1s spectra for (A) carbon black powder and (B) MWCNT powder.


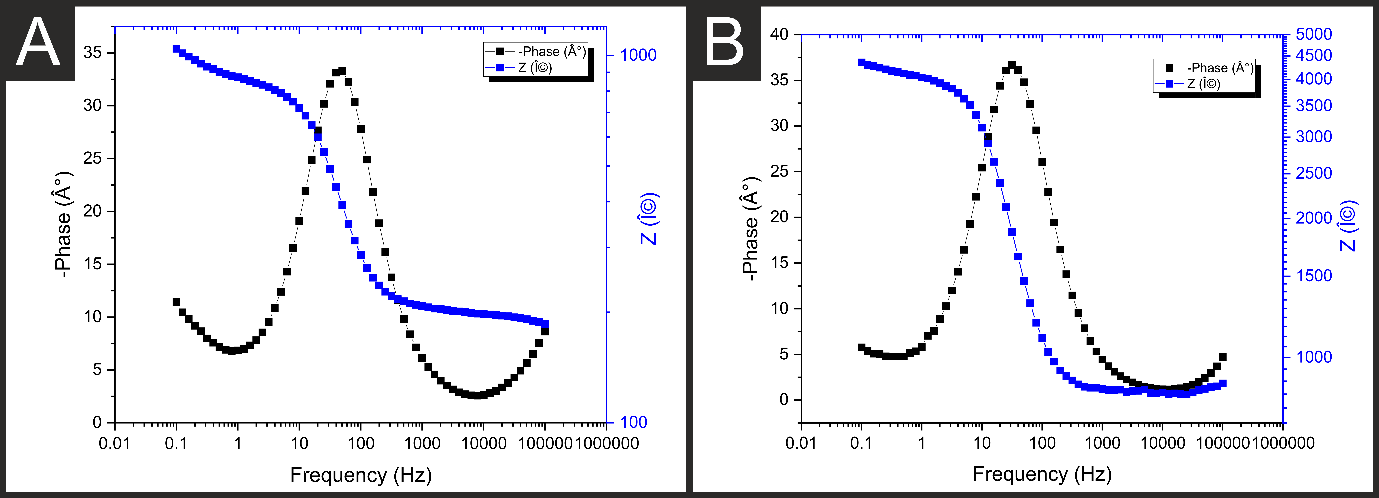
**Figure S3.** EIS Bode plots of [Fe(CN)^­­­^_6_]^4−/3−^ (1 mM in 0.1 M KCl) comparing the (A) MWCNT/CB electrode with the (B) commercial CB/PLA.


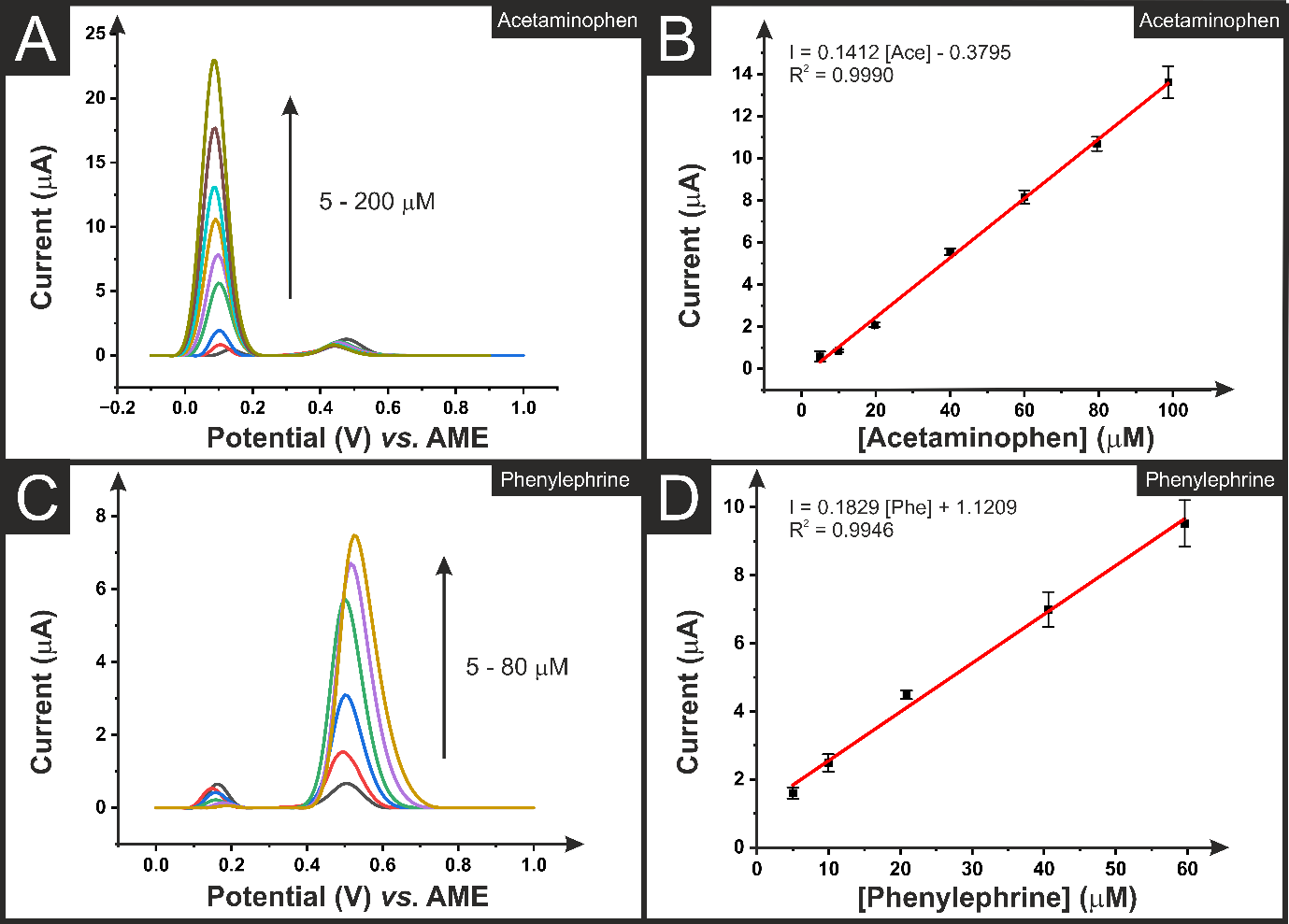
**Figure S1. A)** Differential pulse voltammograms of a fixed concentration of phenylephrine (5 μM) and different concentrations of acetaminophen (5 to 200 μM) in 0.01 M PBS pH 7.4 recorded at MWCNT additively manufactured electrode using commercial additively manufactured electrode as reference and counter electrodes and **B)** the respective calibration plot. **C)** Differential pulse voltammograms of a fixed concentration of acetaminophen (5 μM) and different concentrations of phenylephrine (5 to 80 μM) in 0.01 M PBS pH 7.4 recorded at MWCNT additively manufactured electrode using commercial additively manufactured electrode as reference and counter electrodes and **D)** the respective calibration plot. Step potential: 8 mV. Amplitude: 50 mV.

**Table S1.** Resistance measurements and printability evaluation of the filaments made from different ratios of MWCNT:CB.

| **MWCNT:CB ratio** | **Resistance across 10 cm (Ω)** | **Printability** |
| --- | --- | --- |
| 0:100 | 864 ± 54 | Very good |
| 20:80 | 315 ± 33 | Very good |
| 40:60 | 243 ± 24 | Good |
| 60:40 | 174 ± 93 | Poor |
| 80:20 | 200 ± 55 | Poor |
| 100:0 | 131 ± 87 | Very poor |

**Table S2.** XPS atomic concentration data from the activated and non-activated CB/MWCNT electrodes.

| **Group Assignment** | **Non-activated Electrode %At Concentation** | **Activated Electrode**  **%At Concentation** |
| --- | --- | --- |
| Graphitic | 23.48 | 29.93 |
| C-C/C-H | 34.43 | 35.67 |
| C=O | 21.33 | 15.51 |
| O-C=O | 18.70 | 16.84 |
| π-π* | 2.06 | 2.05 |

**Table S3.** Summary of the fitted values from EIS obtained for electrodes printed from each filament tested (n=3).

| **Element** | **Protopasta** | **CB only** | **MWCNT/CB** |
| --- | --- | --- | --- |
| R1 (Ω) | 650 ± 14 | 222 ± 10 | 115 ± 18 |
| C (x10^-6^ F) | 5.8 ± 0.7 | 26 ± 5 | 23 ± 3 |
| R2 (kΩ) | 2.94 ± 0.62 | 0.47 ± 0.09 | 0.31 ± 0.06 |
| W (x10^-3^ Ω s^-1/2^) | 2.8 ± 1.0 | 2.6 ± 0.3 | 2.7 ± 0.2 |

**Table S4**. Summary of all calibration plots, equations, and R² for acetaminophen and phenylephrine individual and simultaneous analyses.

| Analyte | Range (mM) | Equation | R^2^ |
| --- | --- | --- | --- |
| ACE | 5 - 200 | I = 0.1412[Ace] – 0.3795 | 0.9990 |
| PHE | 5 - 60 | I = 0.1829 [Phe] + 1.1209 | 0.9946 |
| PHE and ACE | 5 - 60 | I = 0.1432 [Phe] + 1.121 and I = 0.0510 [Ace] + 0.9266 | 0.9946 and 0.9897 |
